# Supplementary material for: Functional Characterization of 5-O-Glycosyltranferase Transforming 3-O Anthocyanins into 3,5-O Anthocyanins in Freesia hybrida
Source: Int J Mol Sci. 2025 May 9;26(10):4542. doi: 10.3390/ijms26104542 (PMC12111779; doi:10.3390/ijms26104542)
Supplement: Supplementary file 1 [file ijms-26-04542-s001.zip › ijms-3543434-supplementary.pdf]

Functional characterization of 5-*O*-glycosyltranferase transforming 3-*O* anthocyanins into 3,5-*O* anthocyanins in *Freesia hybrida*

Adnan [1†], Tingting Bao [1,2†], Yicong Pang [1], Ruifang Gao [1], Xiaotong Shan [1], Shirui Zhu [1], Shadrack Kimani [1,3], Xiang Gao [1] and Yueqing Li [1*]

[1] Key Laboratory of Molecular Epigenetics of MOE, Northeast Normal University, Changchun 130024, China
[2] Shenzhen Branch, Guangdong Laboratory of Lingnan Modern Agriculture, Key Laboratory of Synthetic Biology, Ministry of Agriculture and Rural Affairs, Agricultural Genomics Institute at Shenzhen, Chinese Academy of Agricultural Sciences, Shenzhen, 518120, China
[3] School of Pure and Applied Sciences, Karatina University, Karatina, Kenya
* Correspondence: liyq339@126.com.
† These authors contributed equally to this work.

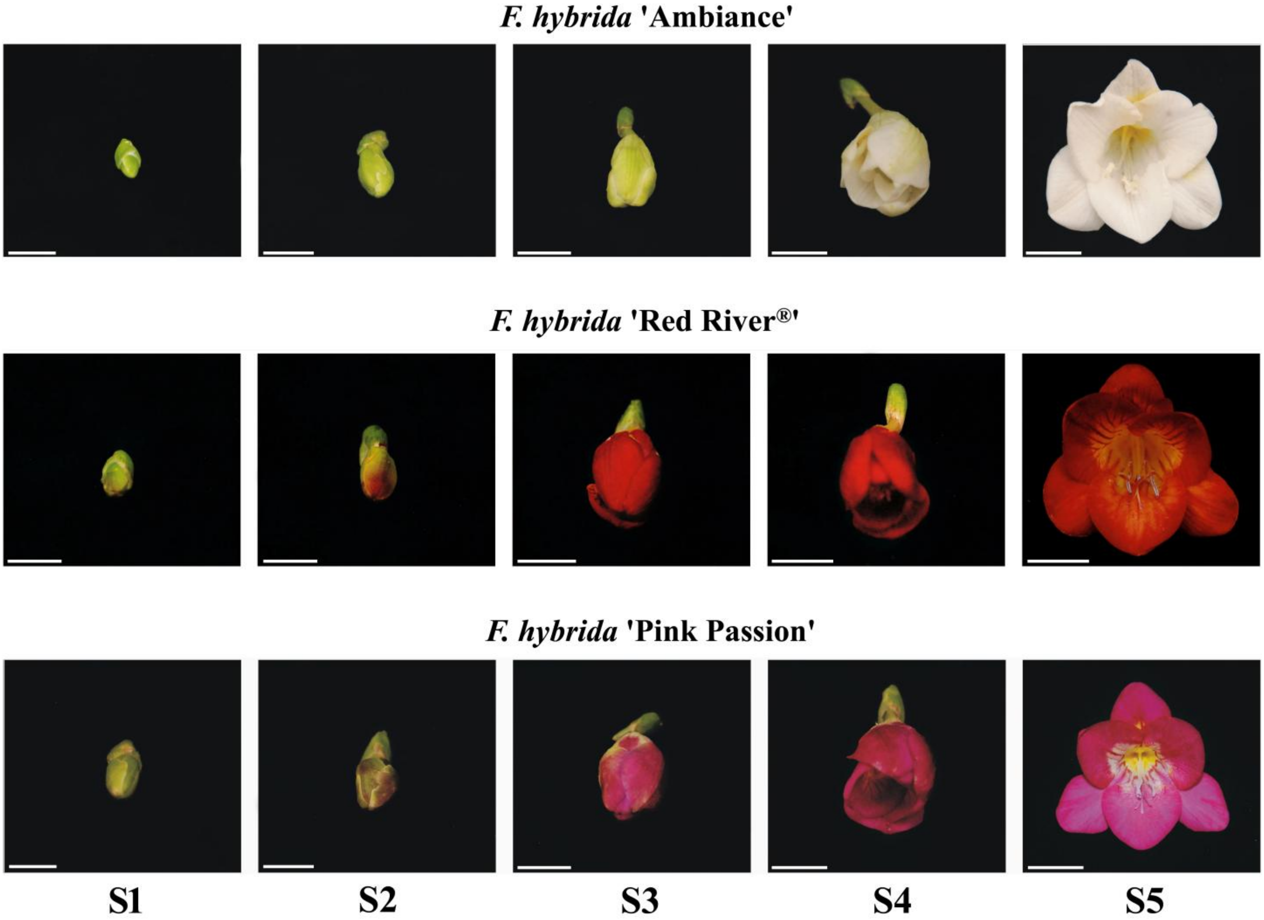

Supplementary Figure S1. Different developmental stages of *F. hybrida* 'Ambiance', *F. hybrida* 'Red River®', and *F. hybrida* 'Pink Passion'.

Five developmental stages from young bud to fully mature flower in each variety. Stage 1: Buds <10 mm in length, unpigmented. Stage 2: Buds 10 – 20 mm in length, slight pigmentation visible. Stage 3: Buds 20 – 30 mm in length, fully pigmented but unopened. Stage 4: Fully pigmented flowers prior to complete opening. Stage 5: Fully opened flowers with mature pigmentation. Bars represent 1.0 cm.

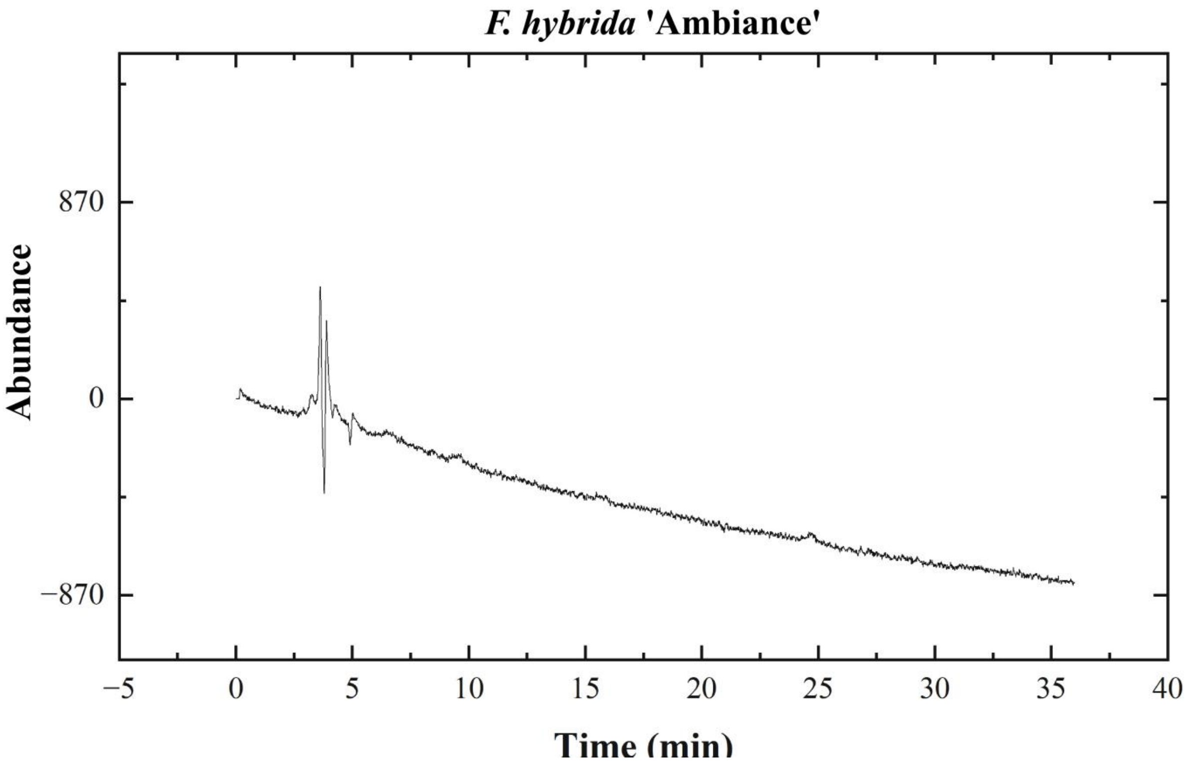

Supplementary Figure S2. **HPLC analysis of anthocyanins in *F. hybrida* 'Ambiance' flower.**
Anthocyanins were extracted by acidic methanol solution from *F. hybrida* 'Ambiance' flowers fully bloomed at the first day and analyzed by HPLC analysis.

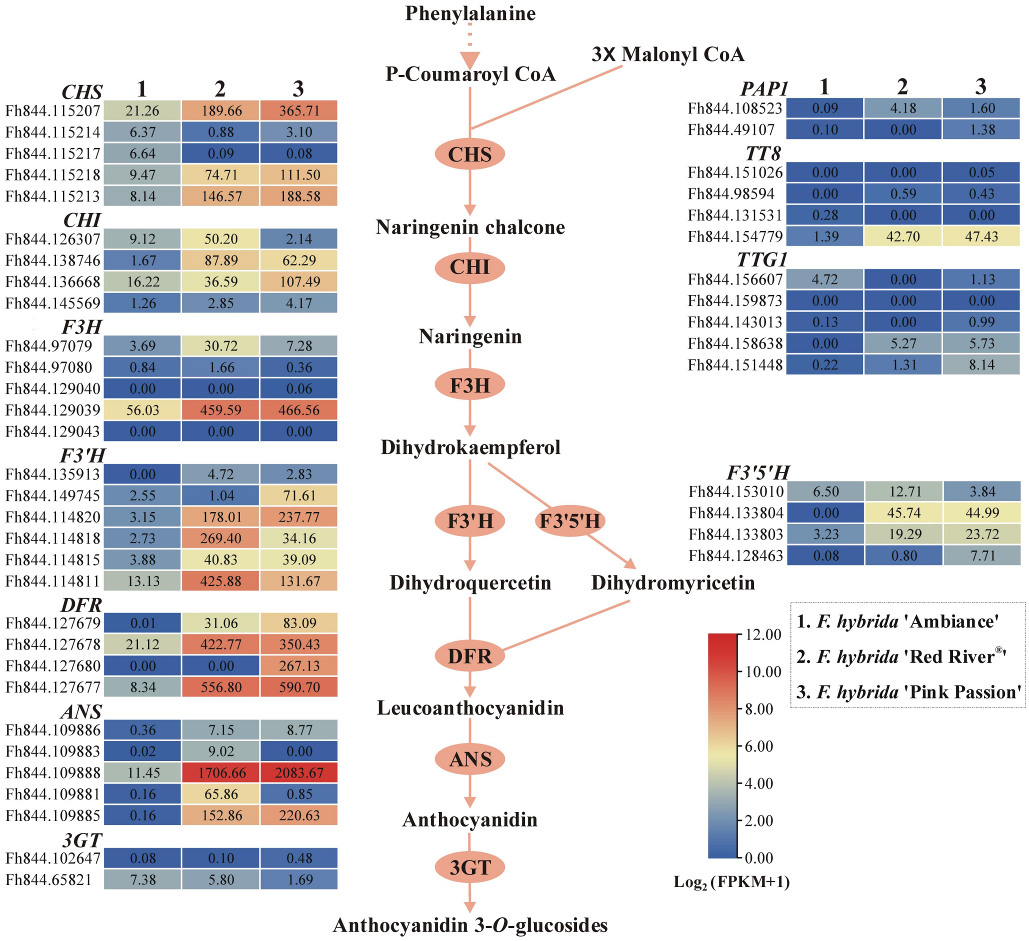

Supplementary Figure S3. Relative FPKM values of anthocyanin biosynthesis-related genes in *F. hybrida* 'Ambiance', 'Red River®', and 'Pink Passion'. Data represented mean FPKM values from three biological replicates.

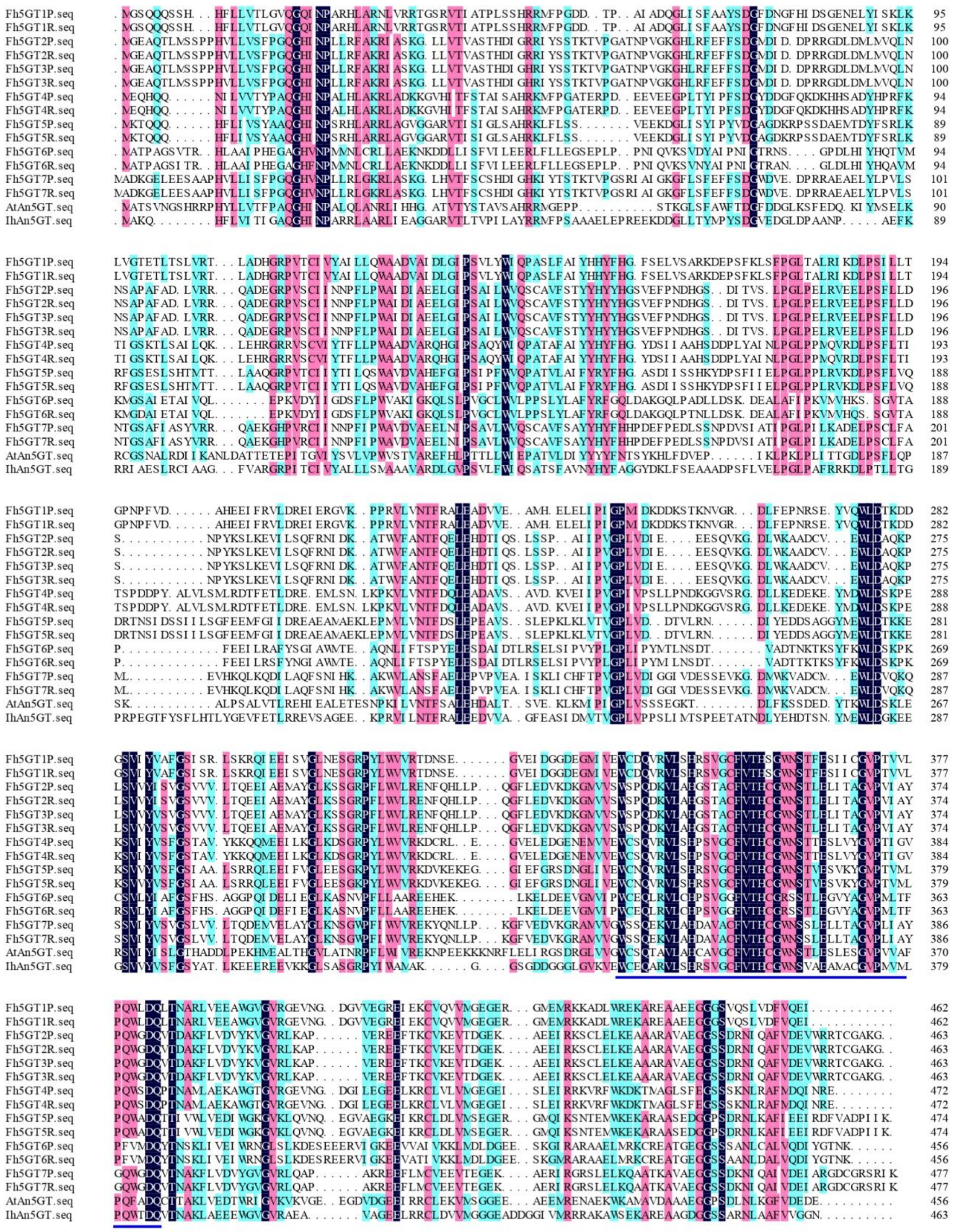

Supplementary Figure S4. **Sequence alignment of Fh5GTs.**

Amino acid alignment of Fh5GTs against IhAn5GT (BAD06874) and AtAn5GT (NP_193146). Gene names ending with "P" indicate genes from *F. hybrida* 'Pink Passion' and those ending with "R" indicate genes from *F. hybrida* 'Red River®'. The identical amino acids were coloured as light blue and pink and the similar ones in dark blue. Solid underline indicates the PSPG domain.

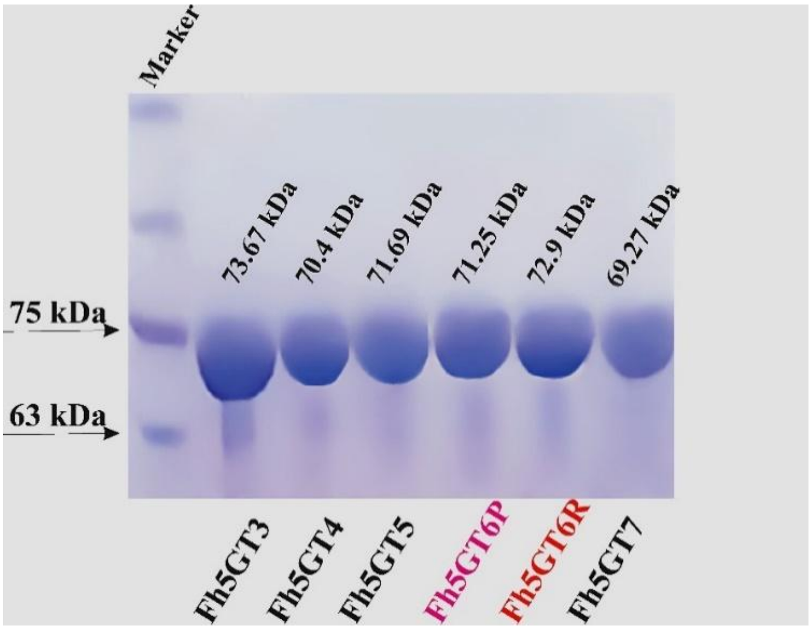

Supplementary Figure S5. **SDS-PAGE analysis of Fh5GTs purified from bacteria.**

The proteins were extracted from bacteria expressing *Fh5GTs* and purified through Ni columns. Routine SDS-PAGE was performed to assess the molecular weights and purification of Fh5GTs.

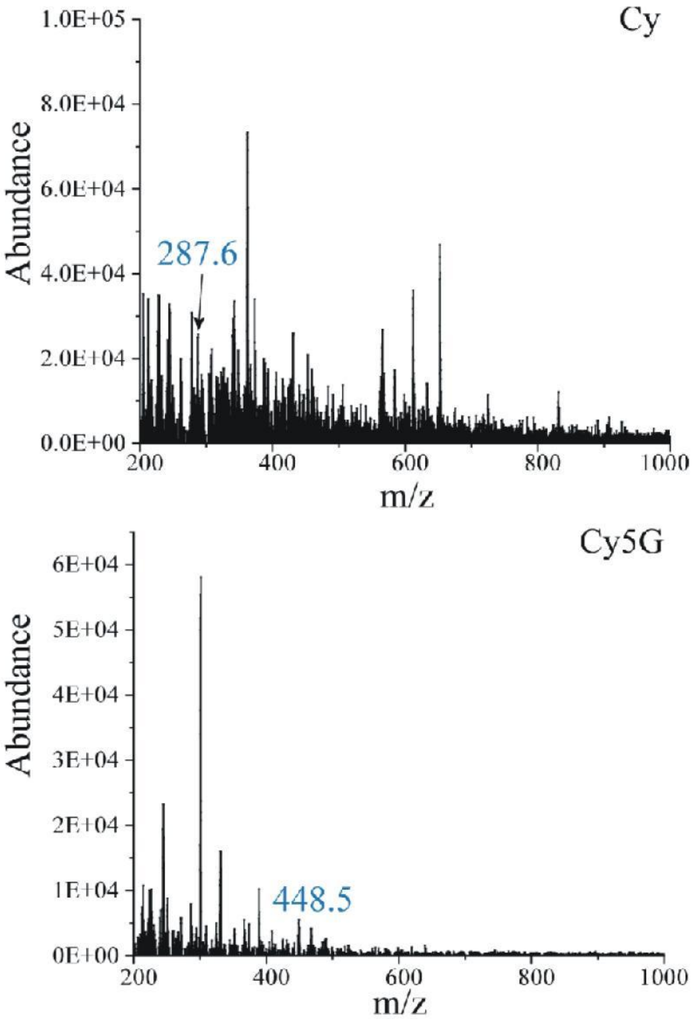

Supplementary Figure S6. Mass spectra analysis of in vitro enzymatic products of Fh5GTs toward cyanidin.

Cyanidin (Cy) and cyanidin 5-*O*-glucoside (Cy5G), with a peak at m/z 287.6 for Cy and a peak at m/z 448.5 Cy5G.

Supplementary Table S1. Primers used in the study

Genes	Upstream(5'-3')	Downstream(5'-3')
Generation of constructs used in heterologous expression in *E. Coli*		
Fh5GT1	TGGCTGATATCGGATCCATGGGAAGTCAGCAGCAGAGTAG	CGACGGAGCTCGAATTCTCAAATCTCTTGCACAAAATCCACA
Fh5GT2	TGGCTGATATCGGATCCATGCAAAGGAAGATCTCGTTCCA	CGACGGAGCTCGAATTCTTAGGACTTTGTTGAGGAGAGCACA
Fh5GT3	TGGCTGATATCGGATCCATGGGAGAAGCTCAAACATTAATG	CGACGGAGCTCGAATTCTCAACCTTTAGCACCACAAGTTCT
Fh5GT4	TGGCTGATATCGGATCCATGGCAAACTCCACTAACAAGCCC	CGACGGAGCTCGAATTCTCACTTGCCTGCCTTGTGTTTATGT
Fh5GT5	TGGCTGATATCGGATCCATGAAGACACAACAGCAACACTTCC	CGACGGAGCTCGAATTCTTACTTTATGATAGGATCCGCAACAA
Fh5GT6	TGGCTGATATCGGATCCATGGCGACACCCGCCGGCAGCGTTA	CGACGGAGCTCGAATTCTCACTTGTTAGTACCATATATGTCT
Fh5GT7	TGGCTGATATCGGATCCATGGCCGACAAGGGAGAGTTAGAAG	CGACGGAGCTCGAATTCTCATGTCTTGATTCTTGAACGACCA
Generation of constructs used in subcellular localization assays		
Fh5GT1	CTGATTACGCTCATATGATGGGAAGTCAGCAGCAGAGTAG	TTGCTCACCATATCGATAATCTCTTGCACAAAATCCACA
Fh5GT2	CTGATTACGCTCATATGATGCAAAGGAAGATCTCGTTCCA	TTGCTCACCATATCGATGGACTTTGTTGAGGAGAGCACA
Fh5GT3	CTGATTACGCTCATATGATGGGAGAAGCTCAAACATTAATG	TTGCTCACCATATCGATACCTTTAGCACCACAAGTTCT
Fh5GT4	CTGATTACGCTCATATGATGGCAAACTCCACTAACAAGCCC	TTGCTCACCATATCGATCTTGCCTGCCTTGTGTTTATGT
Fh5GT5	CTGATTACGCTCATATGATGAAGACACAACAGCAACACTTCC	TTGCTCACCATATCGATCTTTATGATAGGATCCGCAACAA
Fh5GT6	CTGATTACGCTCATATGATGGCGACACCCGCCGGCAGC	TTGCTCACCATATCGATCTTGTTAGTACCATATATGTCT
Fh5GT7	CTGATTACGCTCATATGATGGCGACACCCGCCGGCAGCGTTA	TTGCTCACCATATCGATCTTGTTAGTACCATATATGTCT
RT-qPCR primers		
Fh5GT1	TCGTCGGCACTGAAACCC	ATAGCGACATCGGCAGCC
Fh5GT2	CACCCAAGCCTACCACCG	GCCCTTCTTCGGTGCCAT
Fh5GT3	ACAAGGTTCTCGCCCACG	GATACGCGATCACCGGCA
Fh5GT4	AACAAGCCCCATGCCCTC	GGACGGCTTCGGTGTTGA
Fh5GT5	CGATCGGTCTGTCGGCTC	CATCCGAACTGGGCCGTT
Fh5GT6	CGGTTCGGCCAACTCGAT	CGCCAGAGCTTTGGTGGA
Fh5GT7	GATCGGGAAGCTAAGGGATAAG	TCCGCAAATGATCTCCAAGAG
Generation of constructs used in overexpression assays		
Fh5GT3	TTCTGCCCAAATTCGCGAATGGGAGAAGCTCAAACATTAATG	TTCTGCCCAAATTCGCGATCAACCTTTAGCACCACAAGTTCT
Fh5GT7	AGTTAAAGGCCTCGAGATGGCCGACAAGGGAGAGTTAGAAG	AGTTAAAGGCCTCGAGTCATGTCTTGATTCTTGAACGACCA

Supplementary Table S2. Sequence information of *Fh5GTs* cloned in this study.

Gene name	*Freesia* cultivar	CDS
Fh5GT1	*F. hybrida* 'Pink Passion'<br>*F. hybrida* 'Red River®'	ATGGGAAGTCAGCAGCAGAGTAGCCACCATTTCCTACTAGTCACCCTTGGCGTTCAAGGCCAAATAAACCCGGCCCGCCACCTCGCTCGCAACCT<br>CGTCCGCCGCACCGGCTCTCGTGTCACCATCGCCACCCCTCTCTCCTCCCATCGCCGCATGTTCCCGGGAGACGATACCCCGGCAATCGCAGACC<br>AAGGCCTAATCTCCTTCGCGGCCTACTCCGACGGCTTCGACAACGGCTTCCACATAGACTCTGGCGAAAACGAGCTTTACATTTCCAAACTCAAG<br>CTCGTCGGCACTGAAACCCTAACGAGCCTTGTCCGCACTCTAGCGGACCATGGCCGTCCCGTCACCTGCATTGTCTACGCCATCCTCCTCCAATG<br>GGCTGCCGATGTCGCTATCGACCTCGGCATCCCCTCTGTTCTTTACTGGATCCAGCCTGCCAGTCTGTTTGCTATCTACCATCACTACTTCCATG<br>GATTCTCCGAACTCGTATCGGCCCGTAAAGACGAGCCTTCTTTCAAACTGAGCTTCCCGGGGCTGACTGCGCTTCGAATCAAGGACCTGCCTTCC<br>ATATTGCTCACCGGTCCTAATCCTTTCGTCGATGCACATGAGGAGATATTCCGGGTGCTCGATAGAGAGATAGAGAGAGGCGTCAAGCCGCCGAG<br>AGTGCTAGTGAACACGTTTCGCGCACTCGAGGCCGACGTCGTCGAAGCCATGCATGAGCTCGAACTTATCCCGATCGGTCCAATGATTGACAAGG<br>ACGACAAATCGACCAAGAATGTCGGCCGGGATCTGTTCGAGCCGAATAGAAGCGAGTATGTACAGTGGCTGGACACAAAGGACGATGGTTCGGTG<br>ATATATGTTGCCTTCGGGAGCATTTCACGGCTGTCGAAGAGGCAGATAGAGGAGATATCGGTAGGGCTTAACGAGAGCGGAAGGCCTTATCTTTG<br>GGTGGTGCGAACGGACAACAGCGAAGGAGTCGAGATAGACGGAGGAGACGAGGGGATGATAGTGGAATGGTGTGATCAAGTGAGAGTTCTGTCGC<br>ACCGGTCGGTCGGGTGCTTCGTGACACATAGCGGCTGGAACTCGACATTCGAGAGCATAATTTGTGGCGTGCCGACGGTGATGTTGCCGCAGTGG<br>CTGGACCAGTTGACGAATGCGAGGCTTGTCGAGGAGGCTTGGGGTGTAGGTGTGAGAGGGGAGGTGAATGGGGATGGTGTGGTGGAAGGGAGGGA<br>GATAGAGAAATGTGTACAAGTTGTGATGGGAGAAGGGGAGAGAGGAATGGAGATGAGAAAGAAAGCTGATTTGTGGAGGGAGAAGGCAAGGGAGG<br>CTGCTGAAGAAGGTGGGGGCTCGGTTCAAAGTCTTGTGGATTTTGTGCAAGAGATTTGA
Fh5GT2	*F. hybrida* 'Pink Passion'<br>*F. hybrida* 'Red River®'	ATGCAAAGGAAGATCTCGTTCCAAATGGCTACTCAGCCACATTTCCTTATCGTCTCATTATCTGCTCAGGGCCACATCAATCCAACCCGGCAGCT<br>CGCCCGCCGCCTAGCTCGATTCTGCGGTGCCCGTATCACCATCGCCATCCCCACCCAAGCCTACCACCGCATGTTCTCGTCGTCACCCGACGACA<br>ACAACAACGAACACGACGACGACGACGACAAAGTAAAAGGTCTCGTAACCTACACTGCTTTCTCCGATGGCACCGAAGAAGGGCTCAACCCTCCT<br>ACCGACATGCCGAAAGTCGCGGACCGCGTTGTGGAGAGTCTGACTAGCATAGGAGGTGTGCTTGCAGCCCGGGGCGAGCCGATCACCTGCCTCGT<br>GTACGCTCTCATCATGCCAATGGCTGCACAAGCTGCTCGAAATCTCGGCGTTCCGTCAGTTCTCTTCTGGATCCAACCTGCTTCCGTCTTCGCCA<br>TATATCACCACTACTTCAACAGTGGCTATGCCGAAATCATCGTCGACCACAAAAATGAAGCTTCTTTCTCCGTTGAGCTCCCCGGGTTGGTTCCC<br>CTCCAAATACAGGATCTCCCCTCTTTCCTCACCGACCCAAATCCTGATGATCCATACTATTTCTTCTTCACTCTGTTCGAGCAACTGTTTCAGCT<br>AGTAGCGACCGCGATATCATCCGGATCGAAGCCGACAGTCCTGGTAAACACCTTCCACGAGCTCGAGGCTAGCGTGCTCTCGAGCATCGAAGATG<br>TCGACTTACTCACGGTCGGTCCGTTGGTATCTCCCTCGTCGGGACAAGATCTCTTCGAGCACGACACGAGCGACTACATCGAGTGGCTGGACACG<br>AAGGGGGAGAAGTCGGTGGTGTACGTTTCGTTCGGCAGTCTCGCGGTGCTGTCCAGGGAGCAGATGGAGGAGGTGCTCCGAGGGTTGGAGGAGAG<br>CGAGAGACCGTACATTTGGGTGGTGAGGAGAGACAGTAAAGAGAAAGAGGCTGCAGGCCTCCACGATCGTTACGATGATCGCGACAAGGGGTTGA<br>TAGTGGAGTGGTGCAATCAAGTGAATGTGCTCTCCCACCGGTCTATCGGGTGTTTCGTGACGCACTGCGGGTGGAATTCGACGACGGAGAGTGTG<br>GTCTGCGGCGTGCCGACGGTGATGCTGCCGCAGTGGACGGACCAGAGGACGAATGCGAGGCTGATGGAGGGTATATGGGGATGGGGTTGCGAGC<br>GAAGGCGAGCGAGGAGGGGTTGGTTGAAGCCAAGGAGTTGCAGAGGTGTTTGGATGTGGTGATGGGAGATGAAGATGGAATTGAGATAAGGAGGA<br>GGACAGAAATGTGGAGATATAAGGCACGAGAGGCTGCCATGGAAGGAGGGTCATCAGTTCGAAACATGAGAGCATTTGTGGAGAGATTTGGTGTG<br>CTCTCCTCAACAAAGTCCTAA
Fh5GT3	*F. hybrida* 'Pink Passion'<br>*F. hybrida* 'Red River®'	ATGGGAGAAGCTCAAACATTAATGTCTTCACCCCCTCATGTCCTCCTAGTCTCCTTCCCCGGCCAAGGCCACATTAACCCTCTCCTCCGCTTCGC<br>CAAACGTATCGCCTCCAAAGGCCTCCTTGTCACCGTCGCCTCCACCCACGATATCGGTCGCAGGATCTACAGCTCAACCAAAACCGTACCCGGTG<br>CAACGAACCCCGTCGGCAAAGGCCACCTCCGCTTCGAGTTCTTCTCCGACGGAATGGATATCGACGACCCACGGCGCGGTGACCTTGACATGTTG<br>ATGGTGCAACTTAACAACTCGGCCCCTGCCTTCGCAGACCTTGTCCGCCGTCAGGCTGACGAGGGCCGGCCTGTCTCATGCATCATCAACAACCC<br>TTTCCTCCCTTGGGCGATCGATATCGCGGAGGAGCTCGGCATACCTTCTGCAATCCTCTGGGTACAGTCATGTGCAGTGTTCTCCACGTACTACC<br>ACTACTACCACGGTTCAGTCGAATTCCCCAACGATCACGGCTCCGACATCACCGTGTCCCTCCCGGGATTACCCGAGTTGCGAGTGGAGGAACTT<br>CCTTCCTTCCTCCTCGACTCGAATCCTTACAAAAGCTTGAAGGAAGTTATCCTGTCACAGTTCCGTAACATCGACAAGGCGACATGGGTCTTCGC<br>CAACACCTTCCAGGAGCTCGAGCACGACACGATACAATCCCTCTCCTCCCCTGCCATCATCCCCGTAGGCCCGTTAGTCGATATCGAAGAGGAGT<br>CGCAAGTGAAGGGCGACCTGTGGAAGGCTGCAGACTGCGTGGAGTGGCTCGACGCCCAGAAGCCACTCTCGGTCGTGTACATCTCCGTAGGGAGC<br>GTAGTCGTGCTGACGCAAGAAGAGATCGCCGAGATGGCCTACGGGCTGAAGAGCAGCGGCCGTCCGTTCCTATGGGTGCTTAGAGAGAATTTCCA<br>ACACTTGCTACCACAAGGGTTCTTGGAGGATGTCAAAGATAAGGGAATGGTGGTGAGTTGGAGCCCTCAAGACAAGGTTCTCGCCCACGGCTCGA<br>CCGCCTGCTTCGTGACGCACTGCGGATGGAACTCGACGCTCGAGCTGATTACTGCCGGCGTGCCGGTGATCGCGTATCCGCAATGGGGTGACCAG<br>GTGACCGATGCCAAGTTTCTCGTCGACGTCTACAAAGTCGGGGTGAGGCTCAAGGCTCCGGTGGAGAGAGAAGAGTTTACCAAGTGTGTGAAAGA<br>GGTCACCGACGGCGAGAAGGCGGAGGAGATCAGGAAGAGCTGTTTGGAGTTGAAGGAAGCGGCGGCGAGGGCGGTGGCGGAGGGAGGATCGTCTG

		ATCGTAACATCCAAGCTTTTGTGGATGAGGTATGGAGGAGAACTTGTGGTGCTAAAGGTTGA
Fh5GT4	*F. hybrida* 'Pink Passion'<br>*F. hybrida* 'Red River®'	ATGGCAAACTCCACTAACAAGCCCCATGCCCTCCTCATCACATACCCTCTCCAAGGCCACATCATCCCTTCGGTTCACCTAGCAATGAAACTAGC<br>CTCCAACGGCATCGTCGTCACCTTCGTCAACACCGAAGCCGTCCACCGACAAACTTCCGTAGCAAACGGCTGCTCCGACATCTTCGCCAGTGCCA<br>AAGACGACGGCCTCGACATCCGTTACGAGCTCGTAAGCGACGGGTTGCCCGTCGAGTTCGACCGCTCTCTCGGCCACGATGAGTTCATGGGGTCC<br>CTACTGAACAACCTTTCCGATCACGTTGAGGTGCTTGCACGGAAGTTGTTGCATGCTGACCCCCCGATCACGTGCATCGTCGCCGATACCTTCTT<br>CGTCTGGCCGTCGGCCCTGTCGAAGAAGCTCGGGCTAGTGTACGTGTCGTTCTGGACCGAGCCGGCGCTCGTGTTCACACTGTACTATCACCTTG<br>ACCTGCTAAGGATAAATGGCCATTTTGCCTCTTCTAAAGAACCCCGAAAGGACACGATCACATACATACCGGGCGTCGACACGATCGAGCCTTCC<br>GAACTCATGTCGTACCTCCAAGAATCCGATACGACGTCGGTGTGCCACCGCATCATCTTCAAGGCGTTCGACGACGCGAAGGGTGCAGACTATGT<br>ACTGTGCAACACTGTCCAAGAACTCGAGCCCAGCACGATCGCAGCACTGCATAAGCACAAGCCGTTCTACGCAATCGGTCCGATTTTCCCCGCAG<br>GCTTCACCAGGAGCAGCGTCGCGACGAGCCTATGGGCCGAGTCAGACTGCAGGCAGTGGCTTGATTCCAAGCCTGCCGGCTCTGTCCTGTACGCG<br>TCTTTCGGCAGCTATGCGCACGTTAGCAAGAAGGAACTGGAGGAGATTGCTTGCGGCGTTCGGGACAGCAATGTGAGCTTCCTGTGGGTGTTACG<br>TCCCGACATCGTTAGCTCGGACGAACCGCACCCGCTGCCGATAGGCTTCATGGAGGAGACTAAGGACAGGGGGAAGGTTGTGCAGTGGTGCTGCC<br>AGATCGAGGTGCTATCGCACAAGTCGATAGGAGGGTTCTTGACGCACTGCGGGTGGAACTCGATACTGGAGAGCATATGGAGCGGGGTGCCGTTG<br>CTGTGCTTCCCGCTGCTTACCGACCAATTCACGAACCGCAAGCTCGTGGTTCGGGACTGGAGGATGGGGATCGACCTCGGGGGAGTGGATACGAA<br>TGTGAGTCGTAACGACGTATCGGAGAAAATTTGTAGGTTGATGAAGGGAGAGGAAGGAGATGAGATGAGGGTTGGGGTGAAGGAGGTGAGGAGAG<br>TACTGGAGAGTGCAGTTGGTCCTGATGGTTCTTCCCAGAAGAATTTTGATAGTTTTGTTGCAGATTTGATGAATCATAAGATACATAAACACAAG<br>GCAGGCAAGTGA
Fh5GT5	*F. hybrida* 'Pink Passion'<br>*F. hybrida* 'Red River®'	ATGAAGACACAACAGCAACACTTCCTCATAGTCTCCTATGCAGCCCAAGGCCACATCAACCCTTCCCGCCACCTCGCTCGTCGCCTAGCCGGTGT<br>CGGTGGCGCCAGAGTCACCATCTCGATCGGTCTGTCGGCTCACCGCAAACTGTTCCTCTCCTCTGTTGAAGAAAAAGATGGGCTCATTTCCTACA<br>TTCCCTATGTTGATGGCGCCGGGGACAAACGGCCCAGTTCGGATGCCGAAATGACCGACTACTTCTCGCGGTTGAAGCGATTTGGCTCCGAATCG<br>CTTTCGCATACTATGACCACCTTAGCTGCCCAAGGCCGCCCTGTCACTTGCATCATATACACCATTCTCCAATCATGGGCTGTCGACGTCGCGCA<br>TGAGTTTGGCATCCCTTCCATACCTTTCTGGGTGCAACCCGCCACCGTCTTAGCCATCTTCTACCGCTACTTCCACGGCGCTTCCGATATCATTT<br>CCTCCCACAAGTACGACCCCTCGTTCATTATCGAGTTACCGGGACTCCCGCCTCTCCGTGTCAAAGACCTCCCCTCCTTCCTAGTCCAGGACAGG<br>ACCAACTCTATCGACTCCTCTATCATTCTCTCCGGTTTCGAGGAGATGTTCGGGATCATAGACCGGGAGGCGGAGGCGATGGCGGAGAAACTCGA<br>ACCGATGGTTCTGGTGAACACGTTCGACTCGTTGGAGCCCGAGGCGGTCTCGTCGCTCGAACCGAAGCTGAAGTTGGTGACGGTCGGGCCGCTGG<br>TGGACGACACGGTGCTACGCAACGACATCTACGAGGACGACTCTGCGGGAGGGTACATGGAGTGGCTCGACACCAAGAAGGAGAAGTCGGTAGTG<br>TACGTTTCGTTCGGGAGCATCGCCGCCCTATCGAGACGACAGTTGGAGGAGATTTTTGTAGGGTTGGAAGAGAGCGGGAAGCCGTATCTCTGGGT<br>GGTGAGGAAAGACGTCAAGGAGAAAGAAGGCGGTATCGAGTTCGGCCGTAGCGATAATGGACTAATAGTTGAGTGGTGCAATCAAGTGAGGGTGC<br>TATCGCACCGGTCGGTCGGGTGCTTCGTGACGCATTGCGGGTGGAATTCGACGGTGGAGAGCGTGAAGTACGGCATGCCGACCGTGATGCTGCCG<br>CAATGGGCGGACCAGACGACGATCGTGTGGCTAGTGGAAGACATTTGGGGCAAGGGAGTGAAGCTGCAGGTGAACCAAGAAGGAGTAGCAGAAGG<br>GAAGGAGATAAAGAGGTGTTTGGATTTGGTCATGAGTGAAGGGGAAAGGGGAATGCAGATCAAGAGCAATACAGAGATGTGGAAGGAGAAAGCAA<br>GAGCAGCTTCGGAAGACGGAGGGCCGTCGGATCGGAATCTGAAGGCTTTCATCGAGGAGATCAGAGATTTTGTTGCGGATCCTATCATAAAGTAA
Fh5GT6P	*F. hybrida* 'Pink Passion'	ATGGCGACACCCGCCGGCAGCGTTACCCGGCACTTGGCAGCGATCCCCCATGAGGGTGCCGGCCATGTCAACCCCATGATGAACCTCTGCCGCCT<br>CCTCGCCGAGAAGAACAAGGACGACCTCCTCATAAGCTTCGTCATCCTCGAGGAGCGGCTTTTCCTGCTCGAAGGATCCGAACCTCTCCCCCCGA<br>ACATCCAAGTCAAGTCCGTCGACTACGCGATCCCCAACATCGGCACCAGGAACAGCGGGCCCGACTTGCACATCTACCACCAGACTGTCATGAAG<br>ATGGGGAGTGCAATTGAGACTGCCATTGTGCAATTGGAGCCCAAGGTCGACTACATCATCGGAGACTCGTTCCTGCCGTGGGTTGCCAAGATCGG<br>CAAGCAATTGAGCCTGCCAGTGGGATGCCTGTGGGTGTTGCCGCCGTCGTTGTACTTGGCGTTCTACCGGTTCGGCCAACTCGATGCCAAGGGCC<br>AGTTGCCTGCAGACTTGTTAGATTCTAAAGATGAAGCTCTTGCCTTCATTCCCAAAGTCATGGTCCACAAAAGCTCTGGCGTGACGGCTCCGTTC<br>GAAGAAATCTTAAGAGCATTCTACAGTGGTATCGCTTGGATGACAGAGGCTCAAAACCTCATCTTCACATCTCCTTACGAACTCGAAAGCGATGC<br>CATCGACACCCTCAGGTCCGAATTGTCGATCCCCGTCTACCCATTAGGCCCCTTGATCCCTTACATGACTCTCAATTCCGATACCGTAGCCGACA<br>CCAACAAGACAAAGTCCTACTTCAAGTGGTTAGACTCGAAGCCAAAGTGTTCGGTGCTCTACATCGCCTTCGGTAGCTTCCACTCCGCTGGCGGC<br>CCCCAAATCGACGAGTTGATCGAGGGTCTAAAAGCGAGCAATGTGCCTTTCCTACTGGCTGCTCGCGAAGAGCATGAGAAGTTGAAGGAACTTGA<br>CGAAGAAGTAGGCATGGTGATCCCGTGGTGCGAACAGCTGAGAGTATTGTGCCATCCTTCTGTCGGTGGCTTCGTAACACATTGCGGCAGGAGCT<br>CTACGCTTGAGGGGGTTTATGCCGGCGTGCCGATGCTGACATTCCCTTTTGTGATGGACCAGTATACTAATAGTAAGCTGATTGTGGAGATTTGG<br>AGGAACGGATTGAGTCTGAAGGACGAGTCCGAAGAGGAAAGAGTTATCGGGAAGGAAGAGGTCGTGGCGATCGTGAAGAAGTTGATGGACTTGGA<br>TGGGGAGGAGAGCAAGGGGATAAGGGCGAGGGCTGCCGAGCTTATGCGTAAGTGCCGAGAGGCCACCGGAGAGGGCGGCTCGTCGTCTGCCAACC<br>TTTGTGCTCTTGTTCAAGACATATATGGTACTAACAAGTGA

Fh5GT6R	*F. hybrida* 'Red River®'	ATGGCGACACCCGCCGGCAGCATCACCCGACACTTGGCAGCGATCCCCCACGAGGGTGCCGGCCATTTCAACCCCATGATGAACCTCTGCCGCCT<br>CCTCGCCGAGAAGAACAAGGACGACCTCCTCATAAGCTTCGTCATCCTCGAGGAGAGGCTCTTCCTGCTCGAAGGCTCCGAACCGCTCCCCCCGA<br>ACATCCAAGTCAAGTCTGTCAACTACGCGATCCCCAACATCGGCACCAGGGCCAATGGGCTCGACCTGCACATCTACCACCAGGCTGTCATGAAG<br>ATGGGGGATGCAATTGAGACTGCCATTGTGCAATTGGAGCCCAAGGTCGACTATATCATTGGCGACTCGTTCCTGCCGTGGGTTGCCAAGATCGG<br>CAAGCAATTGAGCCTGCCAGTGGGATGCCTGTGGGTGTTGCCACCGTCGTTGTACTTGGCGTTCTACCGGTTCGGCCAACTCGATGCCAAGGGCC<br>AGTTGCCTACAAACTTGTTAGATTCTAAAGATGAAGCTCTTGCCTTCATTCCCAAAGTCATGGTCCACCAAAGCTCTGGCGTGACGGCTCCGTTT<br>GAAGAAATCTTAAGATCATTCTACAATGGTATCGCTTGGATGACAGAAGCTCAAAACCTGATCTTCACCTCTCCTTACGAACTCGAAAGTGATGC<br>CATCGACACCCTCAGGTCCGAACTCTCGATCCCCGTCTACCCGTTAGGCCCCTTGATCCCTTACATGATTCTCAATTCCGATACCGTAGCCGACA<br>CCACCAAGACAAAGTCCTACTTCAAGTGGTTAGACTCGAAGCCAAAGCGTTCGGTGCTCTACATCGCCTTTGGTAGCTTCCACTCCGCTGGCGGC<br>CCCCAAATCGACGAGTTCATCGAGGGTCTAAAAGCAAGCAATGTGCCTTTTCTACTGGCTGCTCGCGAAGAGCACGAGAAGCTGAAGGAACTTGA<br>CGAAGAAGTAGGCATGGTGATTCCGTGGTGCGAGCAGCTGAGAGTATTGTGCCATCCTTCTGTCGGTGGCTTCGTAACGCATTGCGGCAGGAGCT<br>CTACGCTCGAGGGGGTTTACGCTGGCGTGCCGATGCTGACATTCCCTTTTGTGATGGACCAGTATACGAATAGTAAGCTGATTGTGGAGATTTGG<br>AGGAACGGATTGAGTCTGAAGGACGAGTCCAGAGAGGAAAGAGTTATCGGGAAGGAAGAGGTCGCGACGATTGTGAAGAAGTTGATGGACTTAGA<br>TGGAGAGGAGAGCAAGGGGATGAGGGCGAGGGCTGCCGAGCTTATGCGTAAGTGCCGAGAGGCCACCGGAGAGGGCGGCTCGTCGGCTGCCAACC<br>TTGATGCTCTTGTTCAAGACATATATGGTACTAACAAGTGA
Fh5GT7	*F. hybrida* 'Pink Passion'<br>*F. hybrida* 'Red River®'	ATGAGCACAGGCAGCCACAGCAGTCGGCAGCATGTGGCCATCCTCGCCTTCCCCTTGGCATCGCACCCCGCCAACCTCTTCTCCTTCGCACGTGC<br>CATCGCTGCCGCTGCACCCGACACCACATTCTCCTTCTTCGTCCCCCTCTTCGCTGCCGCCAAACTTCCACCGTCACCGCCGGAGGGCAACCTAC<br>GTGTCCACCAAGTGTTGGACGGCACGCCCGAAGGAGCGGTCCCCGACGCGAATATCCTGCCTTCGATCGAAATGTTCTTGAGGGCGGCACCAGGA<br>AACTTCATAGCCGGGCTGCGGGCAGCAGAGGAGGAGGTGGGGACGAAGGTGGGATGCGTCGTGAGCAATGCGCTTCTGTGGATGGCGGAGGACGT<br>TGCGGAGGAGATCGGAGCAAAGTGTGTGCCACTGTGGACGGCTTCGCATGCCGCCTTGCTAGCTCAAGTGTTCAGTACTGACCAGCAAAGGAAGA<br>GGTTTGGAGCAGAGAAGCAAGCAATGCTGGGGTGGGCAGAGGAGGTGATTGACTGCATACCTGGCTTGTCCTCTCTACGCGCCTGCGACCTCCCC<br>CATAATGTCCTAATGGATCAACACTCCGATATGTCCATCCTCTTCCACGAGGTGGGCAATCGGATCACGAGAGCGACCGCCGTCATCCTCAACAC<br>CTTCCAAGGCCTCGACGCCGCCATCGATGCGGCGCTCGCTTCCACATTCAAGACGACGCTTGCCATTGGCCCCCTCAACCTCCTTTCCCCTCCAT<br>CGCCATTGCAAGCAAGGGACGAGCAATGCCTGTCGTGGCTGGACCGACAAGAGGACGCGACGGTGGCCTATGTCAGCTTCGGCACGATGCCTGCC<br>ACGTTGATGCGTACCCAGGCGACCGAGACAGCCCGTGGCGTCGAGTCTAGCGGGGTGAGGTTCCTGTGGTCCCTACCGGACGACGGGTTGCTCCC<br>TCCCGGGTTCTTGGAGCGGACGGCGGGAAAGGGGCTGGTGGTGCCGTGGGCCCCGCAGGTGAGGGTGCTTGGGCACAAGGCTGTCGGGACCTTCG<br>TGACGCATTGCGGGTGGAATGCGGTGATGGAGAGCATTGCTACCGGGGTGCCGATGGTCTGCGTGCCGTCGTTTGCGGAACAGAAGACGAATGCG<br>AGGATGGTGTCGGCAGAGTGGCGGATCGGGGAGGCGATTCGAGGGGAGACTGTGACGGGGGAGGAGGTGGCGAGGTGCGTAGAGGTGGTGATGAG<br>GGGGGAGGAAGGGAGGAGGATGAGGGAGAGGATCGGGAAGCTAAGGGATAAGGCAGTCGAGGCGATTGGACAGGGCGGGAGCTCGTCGGAGAACT<br>TCAAAGACTCTTGGAGATCATTTGCGGAAATGCTAATGTGTAG
